# COVID-19 and Crossed Eye: A Case Report and Literature Review

**DOI:** 10.7759/cureus.42722

**Published:** 2023-07-31

**Authors:** Hui Di Khor, Pooi Wah Lott, Siti Nor Roha Daman Huri, Sujaya Singh, Tajunisah Iqbal

**Affiliations:** 1 Department of Ophthalmology, Faculty of Medicine, Universiti Malaya Eye Research Centre, Kuala Lumpur, MYS; 2 Department of Ophthalmology, Hospital Sungai Buloh, Selangor, MYS

**Keywords:** severe acute respiratory syndrome coronavirus 2 (sars-cov-2), stroke, covid-19, diplopia, neuro-ophthalmic manifestation

## Abstract

This study aims to report a case of neuro-ophthalmic manifestation in a coronavirus disease 2019 (COVID-19) patient and a literature review of neuro-ophthalmological manifestation in COVID-19 patients. A 57-year-old male presented with headache, giddiness, and sudden onset of diplopia over two days after having a flu-like illness. Clinical examination revealed bilateral bizarre extraocular movement with right lower motor neuron facial nerve palsy. A polymerase chain reaction test for severe acute respiratory syndrome coronavirus 2 (SARS-CoV-2) was positive. Computed tomography (CT) and contrast-enhanced CT of the brain and CT venography were unremarkable. Magnetic resonance imaging (MRI) of the brain, magnetic resonance angiography of the circle of Willis, and MRI of the internal auditory meatus revealed a subacute pontine infarction with no leptomeningeal or cranial nerve enhancement and a normal circle of Willis. The patient received a course of oral prednisolone and clinical symptoms improved gradually. Articles published between December 2019 and June 2022 were included. A total of 23 cases, with 14 male and nine female patients, were summarized. The mean age at presentation was 46.95 years (range = 9-71 years), with the most affected age group ranging from 31 to 70 years (17 of 23 cases, 73.91%). Neuro-ophthalmological symptoms and signs can be isolated or associated with neurological syndromes. The manifestations include optic neuritis, isolated or multiple cranial nerve palsies, acute vision loss, Miller Fisher syndrome, myasthenia gravis, acute disseminated encephalomyelitis, Guillain-Barré syndrome, internuclear ophthalmoplegia, and cerebrovascular events. Diagnosing neuro-ophthalmic complications secondary to SARS-CoV-2 infection is challenging, as there are no pathognomonic symptoms to detect the disease. High clinical suspicion aids in early diagnosis and initiation of treatment may help in relieving the symptoms.

## Introduction

The coronavirus disease 2019 (COVID-19) first emerged in Wuhan, China in late 2019. It spread rapidly across the globe, and the World Health Organization (WHO) declared it a pandemic on March 11, 2020. Severe acute respiratory syndrome coronavirus 2 (SARS-CoV-2) is an enveloped RNA virus that causes this infection [1].

Although acute respiratory distress syndrome is the most important cause of admission for COVID-19 patients, several studies have underlined the presence of neurological symptoms such as confusion, dizziness, impaired consciousness, ataxia, seizure, anosmia, ageusia, vision impairment, and stroke. Neuro-ophthalmologic manifestations of COVID-19 may range from gaze palsy to devastating vision loss [2].

Neurotropic and neuroinvasive capabilities of SARS-CoV-2 have postulated that this virus reaches the central nervous system via neuronal retrograde transmission or hematological spread. Thus, it is no surprise that the virus can manifest as neuro-ophthalmic signs and symptoms. Here, we report a case of neuro-ophthalmic manifestation in a COVID-19 patient.

This article was previously presented as a poster at the 5th ASEAN Ophthalmology Society Virtual Congress 2022 on March 4-5th, 2022.

## Case presentation

A 57-year-old male who was a chronic active smoker with underlying uncontrolled dyslipidemia presented with headache, giddiness, and sudden onset of diplopia over two days. He had a history of high-grade fever and flu-like symptoms at presentation. There was no history of trauma or pre-existing ocular comorbidities. In view of the ongoing COVID-19 pandemic, he was screened for COVID-19 per strict pre-admission protocols. The patient tested positive for COVID-19.

**Figure 1:**
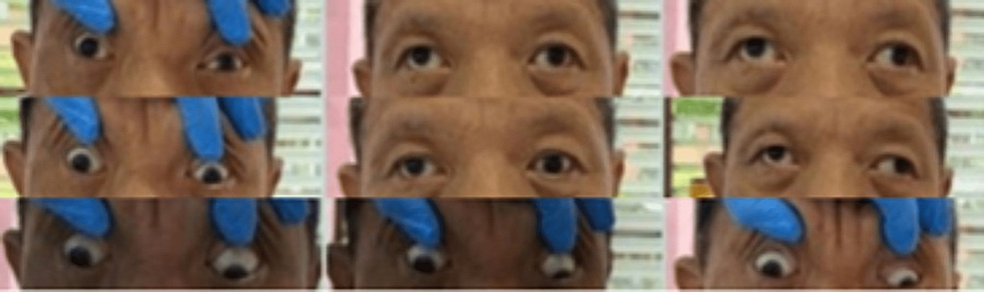
Extraocular movement of bilateral eyes. Extraocular movement limited in all gazes bilaterally.

At presentation, there was bizarre restricted extraocular movement (EOM) in all gazes bilaterally with nystagmus in both lateral gazes (Figure 1). The vision was 6/6 bilaterally. Pupils were equal and reactive. Anterior segments, fundus examinations, and other neurological examinations were unremarkable. There was no relative afferent pupillary defect, ptosis, or facial asymmetry.

An initial plain computed tomography (CT) of the brain was unremarkable. With suspicion of cavernous sinus thrombosis, an urgent contrast-enhanced CT (CECT) of the brain and CT venography (CTV) were done, which revealed no evidence of infarction or cavernous sinus thrombosis.

**Figure 2:**
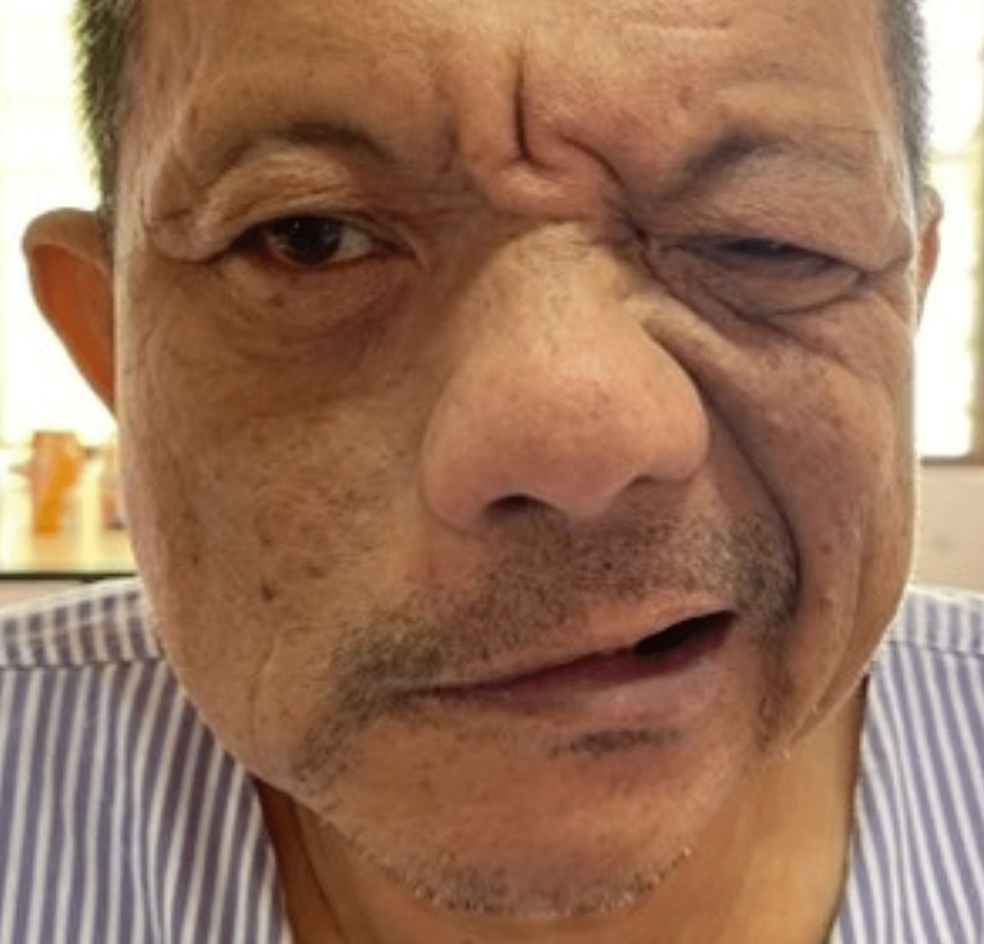
Right lower motor neuron facial nerve palsy.

The patient progressed to develop right lower motor neuron facial nerve palsy, with evidence of slurring of speech, drooling of saliva from the angle of mouth on the right side, inability to close his right eye (Figure 2), and absence of corneal sensation over his right eye on the following day. However, there was no worsening of his EOM.

A comprehensive diagnostic workup was initiated. White blood cell count (11.96 × 10^9^/L) and C-reactive protein (1.3 mg/dL) were elevated. Other laboratory studies, including serum electrolytes, liver function tests, coagulation profile, and random blood sugar were all within normal limits. Lumbar puncture revealed an opening pressure of 11 cmH_2_O with normal biochemistry, cytology, and sterile cultures and negative viral and infective serologies, including reverse transcription polymerase chain reaction for COVID-19. A chest X-ray showed ground-glass opacities of the bilateral lower zones.

The patient was treated for COVID-19-related polyneuritis cranialis by the neuromedical team and completed oral prednisolone 60 mg once daily (1 mg/kg/day) for two weeks.

**Figure 3:**
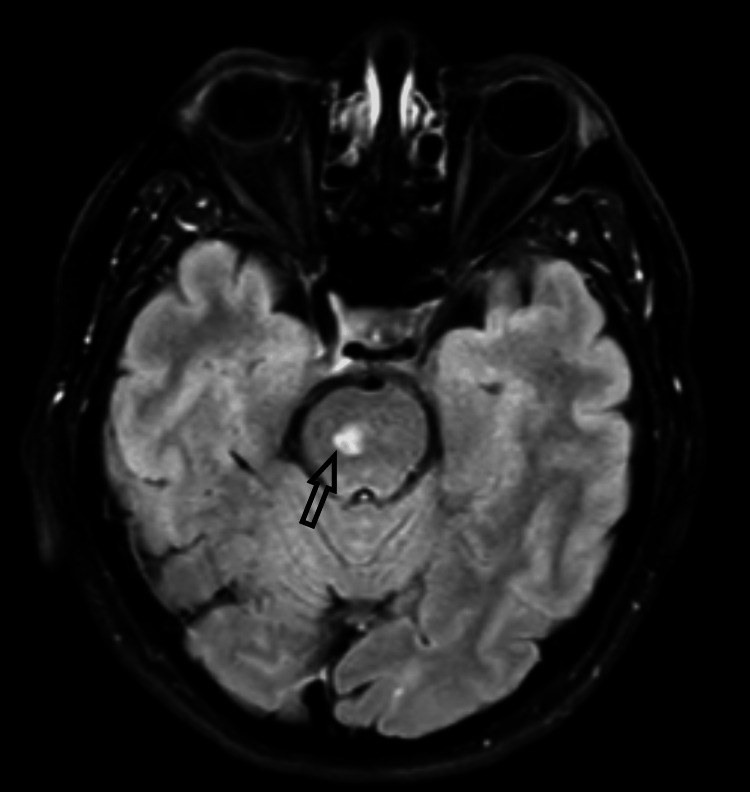
MRI of the brain. MRI of the brain showed subacute right pontine infarction with no leptomeningeal and cranial nerve enhancement and a normal circle of Willis (black arrow).

Outpatient magnetic resonance imaging (MRI) of the brain, magnetic resonance angiography of the circle of Willis, and MRI of the internal auditory meatus conducted three weeks later revealed a subacute pontine infarction with no leptomeningeal or cranial nerve enhancement and a normal circle of Willis (Figure 3).

**Figure 4:**
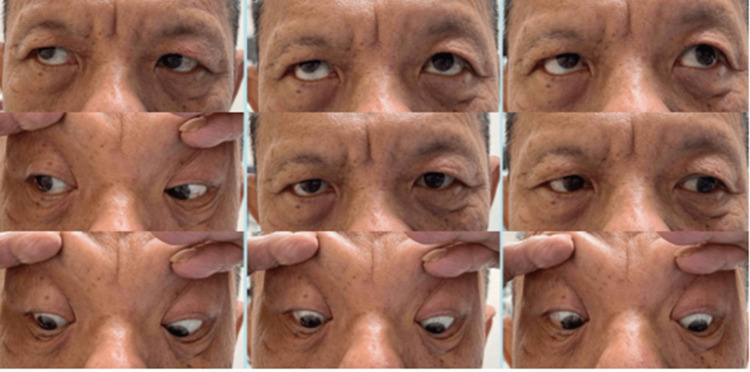
Extraocular movement of bilateral eyes after one month. Improvement of extraocular movement in all gazes.

He was started on aspirin 100 mg tablets once daily and advised on smoking cessation. On follow-up, his ocular symptoms and signs started to improve spontaneously one month after admission (Figure 4). All involved nerve functions were fully restored after a period of five months.

## Discussion

Neuro-ophthalmic complications in COVID-19 patients are increasingly being described in the literature. Regardless of the increasing quantity of published data, none discussed this specific neuro-ophthalmological manifestation of SARS-CoV-2.

A few articles reported that COVID-19 can present as acute vision loss, cranial nerve palsy, optic neuritis, Miller Fisher syndrome, myasthenia gravis, or acute disseminated encephalitis [3], as summarized in Table 1.

**Table 1:** Case summaries of neuro-ophthalmological manifestations in COVID-19 patients.

Authors	Manifestation	Findings
Falcone et al. [10]	Left abducens palsy	A 32-year-old male patient presented with upper respiratory illness and painless acute binocular horizontal diplopia. MRI showed atrophic left lateral rectus muscle
Francis et al. [11]	Left abducens palsy and anosmia	A 69-year-old female patient developed painless binocular diplopia with isolated anosmia as a symptom of COVID-19. No neuroimaging was done
Belghmaidi et al. [12]	Oculomotor nerve palsy	A 24-year-old female patient presented with acute diplopia and left eye strabismus for three days. Magnetic resonance angiography of the brain and orbits revealed no lesions or aneurysmal compression of the third left cranial nerve
Gutiéérrez-Ortiz et al [13]	Miller Fisher syndrome and polyneuritis cranialis	A 50-year-old male presented with right internuclear ophthalmospasm, right fascicular oculomotor palsy, ataxia, and areflexia, followed by a few days of typical COVID-19 symptoms. Plain CT of the brain was unremarkable
		A 39-year-old male presented with bilateral abducens palsy and areflexia, followed by a few days of COVID-19 infection. Plain CT of the brain was unremarkable
Reyes-Bueno et al. [14]	Miller Fisher syndrome	A 51-year-old female patient presented with bilateral lower limb weakness and binocular horizontal diplopia. MFS was diagnosed two weeks after COVID-19 infection. Neuroimaging studies were normal
Restivo et al. [15]	Myasthenia gravis	Three patients developed symptoms suggestive of myasthenia gravis. Electromyography with repetitive stimulation showed decrement and the concentration of acetylcholine was elevated
Dinkin et al [16]	Presumed Miller Fisher syndrome	A 36-year-old man with a history of infantile strabismus presented with left ptosis, diplopia, and bilateral distal leg paraesthesia. MRI revealed enhancement, T2 hyperintensity, and enlargement of the left oculomotor nerve. A ganglioside antibody panel was negative
	Right abducens nerve palsy	A 71-year-old woman with hypertension presented with painless diplopia on waking two days prior and could not abduct her right eye. MRI showed enhancement of the optic nerve sheaths and posterior Tenon capsules
Juliao Caamañño et al. [17]	Guillain Barré syndrome	A 61-year-old male noted liquid dripping on his right facial commissure on day 10 of COVID-19 infection. On the day after, symptoms progressed toward bilateral facial nerve palsy with unresponsive blink reflex on both eyes, otherwise no other systemic neurological symptoms. Neuroimaging was unremarkable
Capponi et al. [18]	Right abducens nerve palsy	A nine-year-old girl with right abducens nerve palsy on day three of COVID-19 infection with no evidence of other neurological disease on neuroimaging
Daniel et al. [19]	Acute bilateral occipital territorial ischemic infarct	A 61-year-old man with underlying type 2 diabetes mellitus presented with bilateral, sudden, and painless loss of vision for two days. Visual acuity of bilateral eyes is the perception of light with no relative afferent pupillary defect. Anterior and fundus examinations were unremarkable. CT brain revealed loss of gray-white matter differentiation compatible with cytotoxic edema in the bilateral occipital polar regions without hemorrhage
	Acute right middle cerebral artery infarct	A 34-year-old woman with underlying systemic lupus erythematosus, hypertension, end-stage renal disease, chronic obstructive pulmonary disease, and a history of stroke without any functional deficits (good central vision) presented with sudden bilateral painless loss of vision for two days. Visual acuity of bilateral eyes is the perception of light. The relative afferent pupillary defect was negative. Fundus examination showed bilateral pale optic discs. MRA of the brain revealed an occlusion of the M2 branches of the right middle cerebral artery, with otherwise normal flow in the other major intracranial arterial branches
Bondira et al. [20]	Visual field deficits consistent with stroke	A 68-year-old woman presented with difficulty in reading after one-month hospitalization for COVID-19. Automated visual fields revealed a right homonymous hemianopia and a subtle left superior homonymous quadrantanopia. MRI of the brain with and without contrast revealed a large subacute to chronic infarct in the left occipitotemporal region and a smaller right occipital infarct

Cranial nerve palsies in COVID-19 may occur as mono-neuropathy or polyneuritis cranialis, with or without peripheral or central nervous system involvement. In a retrospective case series conducted in Wuhan, China involving 214 patients infected with the virus, 36.4% had nervous system signs and symptoms, including headache, giddiness, hypogeusia, hyposmia, and ischemic or hemorrhagic stroke [4].

In a single-center, retrospective study in Wuhan, China conducted by Li et al. of 219 patients with confirmed SARS-CoV-2, 11 (5.0%) developed new-onset stroke following COVID-19 infection [5]. Of these patients, 10 (90.9%) patients were diagnosed with acute ischemic stroke and one (9.1%) had intracerebral hemorrhage.

A notable number of patients with COVID-19 have experienced ischemic strokes, although the relationship between the virus and cerebrovascular events is not yet well understood [6]. The possible mechanisms include viral neurotropism, endothelial dysfunction, coagulopathy, and inflammation [7].

A postmortem histological analysis by Varga et al. of various organs demonstrated the presence of SARS-CoV-2 viral elements within endothelial cells and evidence of endotheliosis and inflammatory cell death, which subsequently lead to organ ischemia, tissue edema, and a procoagulant state [8]. The procoagulant state that occurs during COVID-19 infections also predisposes patients to cerebral vascular accidents (CVAs) which occur at an increased risk in those with underlying comorbidities [9].

In view of these proposed mechanisms, we believe that our patient has a higher prevalence of getting CVA in association with COVID-19 infection as he is an active chronic smoker with underlying uncontrolled dyslipidemia. Smoking-induced hypercoagulation in human plasma contributes to thrombus formation, and it can directly injure endothelial cells, which contributes to atheroma formation, augmenting the risk of thrombotic occlusive events.

## Conclusions

Understanding the pathophysiology, clinical manifestation, and natural history of COVID-19 is limited. A direct causal link between SARS-CoV-2 and its neurological deficits was difficult to ascertain. Hence, clinicians should be aware of the possibility of the association between COVID-19 and neuro-ophthalmic disease.
